# PERCC1‐associated enteropathy: Diagnostic challenges and enteral autonomy achieved with teduglutide

**DOI:** 10.1002/jpr3.70144

**Published:** 2026-01-21

**Authors:** Angela Tran, Vivien Nguyen, Phuong Huynh

**Affiliations:** ^1^ Pediatrics, UCSF Benioff Children's Hospital Oakland Oakland California USA; ^2^ Pediatric Gastroenterology, UCSF Benioff Children's Hospital Oakland Oakland California USA

**Keywords:** chronic diarrhea, congenital enteropathy, GLP‐2, intestinal failure, parenteral nutrition

## Abstract

Congenital diarrheas and enteropathies (CODE) are rare inherited disorders characterized by early‐onset intractable diarrhea. Though progress has been made in elucidating the genetic basis of CODE, much remains to be discovered. Another challenge is the lack of curative therapies—treatment is primarily supportive including enteral and parenteral nutrition, and at times, intestinal transplant. We report a 3‐year‐old with intractable diarrhea and failure to thrive in infancy. Whole exome sequencing revealed uniparental disomy in chromosome 16. Whole genome sequencing later identified a novel point mutation in PERCC1 (proline and glutamate‐rich protein with a coiled coil domain 1), a previously unannotated reading frame flanking the regulatory sequence of the “intestine‐critical region”, linked to enteroendocrine cell function and congenital enteropathy. Despite interventions to ameliorate malabsorption, the patient was dependent on partial parenteral nutrition secondary to profuse osmotic diarrhea, for two consecutive years. He was weaned off parenteral nutrition after starting teduglutide.

## 1 INTRODUCTION

Congenital diarrhea and enteropathies (CODE) are a rare group of conditions presenting with neonatal or infantile diarrhea. The evaluation for CODE may include lab tests, diagnostic imaging, as well as endoscopy with histology, but evaluating for underlying mutations has become standard of care.^1^ Whole exome sequencing identifies mutations in coding genes, but there are limitations, such as noncoding genes and unannotated genes that may require whole genome sequencing.

PERCC1 (proline and glutamate‐rich protein with a coiled coil domain 1) is one such previously unannotated coding gene flanking the intestine‐critical region (ICR) on chromosome 16. Its importance was discovered in the genome of patients with congenital diarrhea who had homozygous deletions of the ICR. Mouse models showed that targeted deletion of PERCC1 led to reduced body weight and intestinal dysfunction, reversible by PERCC1 transgene insertion. Ribonucleic acid sequencing further demonstrated that mice with the disrupted gene had reduced gastrin, somatostatin and ghrelin expression from enteroendocrine cells (EEC), and low serum gastrin,^2^ implicating PERCC1 in normal EEC function.

We report the case of a 3‐year‐old with PERCC1‐associated congenital enteropathy, initially missed on whole exome sequencing. Due to an area of uniparental disomy of chromosome 16, also known as the intestine‐critical region, the patient underwent whole genome sequencing, which revealed a novel point mutation in PERCC1. The patient began teduglutide for intestinal failure and was able to wean off of total parenteral nutrition (TPN) and gain enteral autonomy after 2 months.

## 2 CASE REPORT

A full‐term Caucasian male was born appropriate for gestational age. At 18 days, he presented for diarrhea and emesis, weighing 8% below birth weight despite avid breastfeeding supplementation with standard infant formula. Physical exam was normal. Diarrhea persisted despite maternal elimination of dairy and soy, and trials of partially and extensively hydrolyzed formulas, and amino acid‐based formulas.

Symptoms improved briefly with bowel rest. Labs showed low stool pH of 4.0 and high stool osmotic gap of 169.2 mOsm/kg, suggesting diet‐induced diarrhea and carbohydrate malabsorption. However, normal stool reducing substances while receiving an extensively hydrolyzed formula indicated a mixed etiology. Non‐reducing substances were not examined. Additional labs revealed normal elastase on formed stool, elevated fecal fat, normal calprotectin, negative gastroenteric pathogen panel and bacterial culture, normal serum zinc, normal serum lipids, normal fecal alpha‐1 antitrypsin, negative workup for inborn errors of metabolism, undetectable immunoglobulins IgM, IgA, and IgE, and normal IgG. Upper gastrointestinal series and small bowel follow through showed rapid transit but no anatomic abnormalities. Endoscopy was unremarkable. Disaccharidase assay demonstrated low maltase and sucrase activity.

Given concern for congenital sucrase‐isomaltase deficiency, a carbohydrate‐free formula was started. However, after initial tolerance, diarrhea worsened and led to dehydration with metabolic acidosis. Malabsorption and intestinal failure prompted initiation of TPN at 1 month of age. At 2 months, a central venous catheter was placed for long term nutrition. He trialed amino acid‐based formulas and started solids at 4 months while receiving TPN, with fluctuating improvement in diarrhea. Medications included loperamide, which helped decrease his stool frequency, and Creon, which was started after a repeat pancreatic elastase on semi‐formed stool revealed moderate insufficiency and was used intermittently with inconsistent effect. He returned to birth weight z‐score at 4 months, but required gastrostomy tube placement at 2 years of age due to intermittent diarrhea and for nutrition supplementation in addition to TPN.

Genetic work‐up, including a primary immunodeficiency panel, congenital diarrhea genes, single nucleotide polymorphism microarray, and whole exome sequencing, demonstrated increased total homozygosity in chromosome 16, consistent with maternal uniparental disomy. Literature review revealed reports of genomic deletions of the intestine‐critical region of chromosome 16 that resulted in congenital enteropathy.^2^ This prompted whole genome sequencing through the undiagnosed disease network (UDN). At 27 months, he was diagnosed with PERCC1 mutation (16:1432941 C > G; NM_001365310.2:c.348 C > G; NP_001352239.1:p.Tyr116Ter).

At 28 months of age, he began teduglutide, and within 1 week, he had significant improvement in his stool frequency and consistency. After 2 months, he discontinued TPN, and after 6 months of teduglutide, he no longer required gastrostomy tube feeds. He now takes solids and formula orally, teduglutide daily, and loperamide, as needed. Creon was discontinued after repeat pancreatic elastase levels while on teduglutide were normal. Formula was transitioned from an amino acid‐based to peptide‐based formula with normal stools. Weight was following the 80th percentile curve (z‐score 0.84) with length following the 90th percentile trajectory (z‐score 1.44) and body mass index at 34th percentile (z‐score −0.4) at last assessment.

## 3 DISCUSSION

We present a case of a patient with congenital diarrhea of unclear etiology who was diagnosed after whole genome sequencing. While genetic testing is a standard component of the CODE workup, it is often limited by whole exome sequencing. This confines the analysis to annotated and coding genes, whereas whole genome sequencing can uncover mutations in both unannotated and noncoding genes. With more awareness, PERCC1 could also be included in congenital diarrhea and enteropathy panels. Streamlining the diagnostic process for patients with ambiguous clinical workup can enable earlier treatment.

There have only been 11 reports of PERCC1‐associated congenital enteropathy,^2^, ^3^ and our case is notable as the first to document this specific point mutation. It is also the second case where teduglutide reversed intestinal failure. The first patient was Patient A, initially reported by Marek‐Yagel et al.^3^ and described by Stenke et al. when teduglutide use allowed for enteral autonomy.^4^ Patient A was on teduglutide for 1 week prior to discontinuing parenteral nutrition, which they were receiving twice weekly at the time, making up 32% of the weekly caloric requirement.^4^ Our patient was on teduglutide for 2 months prior to discontinuing parenteral nutrition, which he was receiving 4 days a week, making up 38% of the weekly caloric requirement. The remaining 62% of weekly caloric requirement was received enterally. A further comparison of the clinical features, work up and outcomes between our patient and Patient A is shown in Table 1.

**Table 1:** Comparison of two unrelated patients with PERCC1 mutations who responded to teduglutide therapy (adapted from Marek‐Yagel et al.^3^ with data compiled from Stenke et al.^4^).

	Our patient	Patient A^3^, ^4^
Demographic and clinical presentation		
Sex	Male	Male
Presenting symptom(s)	Diarrhea, vomiting	Diarrhea, hypernatremic dehydration
Age at presentation	18 days old	3 weeks old
Age at diagnosis	2 years 3 months	11 years
Age starting teduglutide	2 years 4 months	14 years 7 months
Current age (years)	4 years 2 months	16 years 8 months
Comorbidities	T‐cell lymphopenia, Von Willebrand disease	G6PD deficiency, Glomuvenous malformation right thigh and scalp
Variant in PERCC1	c.348 C > G	c.390 C > G
Laboratory data		
Stool studies		
Sodium (mEq/L)	28.4	9
Potassium (mEq/L)	32	12
Chloride (mEq/L)	8	297
Osmotic gap	169.2	248
Reducing substances	Normal	Abnormal
Elastase (μg/g)	>800	430
Fecal fat (g/day)	Abnormal	298 (3‐day fecal fat collection)
Alpha‐1‐antitrypsin (mg/g)	Normal	Normal
Calprotectin (μg/g)	Normal	Not done
Stool culture	Negative	Negative
Gastrointestinal viral panel PCR	Negative	Negative
Serum studies		
Zinc (μg/dL)	Elevated	Normal
Low‐density lipoprotein (mg/dL)	Normal	Not done
Transferrin (mg/dL)	Low	Normal
Lactate (mmol/L)	Elevated	Normal
Pyruvate (mmol/L)	Normal	Normal
Anti‐enterocyte antibody	Not done	Negative
Amino acid profile	Normal	Normal
Enteroendocrine hormone profile		
Pancreatic polypeptide	Not done	Negligible
Gastrin	Not done	Negligible
Peptide YY 1‐36 and 3‐36	Not done	Negligible
Ghrelin	Not done	Negligible
Glucagon‐like peptide 2	Not done	Negligible
Urine		
Organic acid profile	Normal	Normal
Catecholamines	Not done	Normal
Purines and pyrimidines	Not done	Normal
Histopathology		
Upper endoscopy and colonoscopy	Normal	Normal
PAS/CD10 stain	Normal	Normal
Chromogranin A/Synaptophysin	Normal	Normal
Disaccharidases (μM/min/g protein)	Lactase – 23.2	Not done
	Sucrase – 18.1	
	Maltase – 58.7	
	Palantinase – 6.4	
Liver biopsy	Not done	Macro‐ and microvesicular steatosis (on parenteral nutrition)
Sweat chloride (mmol/L)	Not done	13
Genetic and molecular testing		
Mitochondrial DNA sequencing	Not done	Normal
Whole exome sequencing	Negative	Negative
Whole genome sequencing	Homozygous c.348 C > G stop gain variant	Not done
PCR and Sanger sequencing of the entire coding region and intron boundaries of PERCC1	Not done	Homozygous c.390 C > G stop gain variant
Management and outcomes		
Total duration of parenteral nutrition	2 years 4 months	14 years 7 months
Parenteral nutrition as a percentage of weekly caloric requirement (%)	38	32
Current teduglutide dose	0.04 mg/kg daily	0.015 mg/kg daily
Side effects of teduglutide	Mild abdominal discomfort and nausea after injection	Mild nausea after injection
Current diet	Regular diet	Regular diet
Nutrition supplementation	Peptide formula	Peptide formula
Stools per 24 h	1–2	1–2

Abbreviations: CD, cluster of differentiation; DNA, deoxyribonucleic acid; GP6D, glucose‐6‐phosphate dehydrogenase; PAS, periodic acid‐Schiff; PCR, polymerase chain reaction; PERCC1, proline and glutamate‐rich protein with a coiled coil domain 1.

Teduglutide, an analog of glucagon‐like peptide 2 (GLP‐2), is a trophic gut hormone that helps support intestinal growth and function. It has been shown to increase small intestinal villi height and crypt depth,^5^ which may improve absorption in cases of congenital diarrhea and enteropathy. In our patient with PERCC1 mutation, we suspect that teduglutide is partially compensating for enteroendocrine cell dysfunction and supporting mucosal adaptation by increasing surface area, which helps enhance fluid and nutrient absorption.

Treatment for CODE often includes supportive care with enteral and parenteral nutrition to meet the nutrition and fluid requirements needed to support normal growth and development. However, with early diagnosis and the use of teduglutide, it may be possible to reduce or even eliminate the need for parenteral nutrition.

## 4 CONCLUSION

Teduglutide is a GLP‐2 analog that enabled our patient to achieve enteral autonomy and to be liberated from a restrictive diet. It is indicated for short bowel syndrome but has also reversed intestinal failure in two documented cases with PERCC1‐associated congenital enteropathy.^4^ GLP‐2 promotes mucosal health by increasing crypt cell proliferation and enhancing nutrient absorption. Further studies are essential to explore the potential applications of GLP‐2 analogs in CODE, especially in the context of enteroendocrine disorders.

## CONFLICT OF INTEREST STATEMENT

The authors declare no conflicts of interest.

## ETHICS STATEMENT

Parental informed consent was obtained for the publication of the case details.

## DISCLOSURE

Since teduglutide is FDA‐approved only for short bowel syndrome, its use in this case of intestinal failure represents an off‐label application. We disclose that teduglutide is not currently labeled for this indication.
